# The influence of exercise during pregnancy on racial/ethnic health disparities and birth outcomes

**DOI:** 10.1186/s12884-021-03717-5

**Published:** 2021-03-26

**Authors:** Madigan J. Raper, Samantha McDonald, Carol Johnston, Christy Isler, Edward Newton, Devon Kuehn, David Collier, Nicholas T. Broskey, Adrienne Muldrow, Linda E. May

**Affiliations:** 1 Department of Business, East Carolina University (ECU), 1851 MacGregor Downs Rd, MS#701, Greenville, NC 27834 USA; 2 Department of Kinesiology, ECU, Greenville, NC USA; 3 Department of Kinesiology and Recreation, Illinois State University, Normal, IL USA; 4 Department of Human Development and Family Science, ECU, Greenville, NC USA; 5 Department of Obstetrics and Gynecology, ECU, Greenville, NC USA; 6 Department of Pediatrics, ECU, Greenville, NC USA; 7 Department of Communication, ECU, Greenville, NC USA; 8 Department of Foundational Sciences and Research, ECU, Greenville, NC USA

**Keywords:** Race, Health disparities, Maternal, Exercise

## Abstract

**Background:**

Non-Hispanic black (NHB) pregnant women disproportionately experience adverse birth outcomes compared to Non-Hispanic white (NHW) pregnant women. The positive effects of prenatal exercise on maternal and neonatal health may mitigate these disparities. This study evaluated the influence of prenatal exercise on racial/ethnic disparities in gestational age (GA), birthweight (BW), and risks of preterm birth (PTB), cesarean section (CS), and low-birthweight (LBW) neonates.

**Methods:**

This study performed a secondary data analysis using data from a 24-week, two-arm exercise intervention trial (ENHANCED by Mom). Women with singleton pregnancies (< 16 weeks), aged 18–40 years, BMI between 18.5–34.99 kg/m^2^, and no preexisting health conditions were eligible. The aerobic exercisers (EX) participated in 150 min of moderate-intensity weekly exercise while non-exercising controls (CON) attended low-intensity stretching/breathing sessions. Data on GA, PTB (< 37 weeks), BW, LBW (< 2.5 kg), and delivery mode were collected. Poisson, median and linear regressions were performed.

**Results:**

Participants with complete data (*n* = 125) were eligible for analyses (EX: *n* = 58, CON: *n* = 67). NHB pregnant women delivered lighter neonates (β = − 0.43 kg, 95% CI: − 0.68, − 0.18, *p* = 0.001). After adjusting for prenatal exercise, racial/ethnic disparities in BW were reduced (β = − 0.39 kg, 95% CI: − 0.65, − 0.13, *p* = 0.004). Prenatal exercise reduced borderline significant racial/ethnic disparities in PTB (*p* = 0.053) and GA (*p* = 0.07) with no effects found for CS and LBW.

**Conclusions:**

The findings of this study demonstrate that prenatal exercise may attenuate the racial/ethnic disparities observed in neonatal BW, and possibly GA and PTB. Larger, diverse samples and inclusion of maternal biomarkers (e.g., cytokines) are encouraged to further evaluate these relationships.

## Introduction

Despite recent healthcare reform in the US to minimize racial/ethnic disparities among pregnant women, the prevalence of adverse pregnancy outcomes remains higher among select minority groups, specifically, Non-Hispanic black (NHB) pregnant women [1]. Recent national birth record data shows that 14.1% of NHB pregnant women deliver preterm (< 37 weeks of gestation) compared to 9.0% among Non-Hispanic white (NHB) pregnant women [2]. Greater proportions of NHB pregnant women deliver via cesarean section (36.1% vs 30.8%) and deliver more very-low- (2.9% vs 1.0%) & low-birthweight (14.1% vs 6.9%) neonates compared NHW pregnant women [2]. These adverse delivery and birth outcomes may negatively affect the health of the neonate at birth. Consequently, consistent evidence shows that the well-being of neonates at birth is a strong indicator of future health trajectory, with poorer well-being at birth predisposing neonates to future health complications including obesity, type 2 diabetes mellitus, respiratory infections, cognitive delays, etc. [3, 4]. Therefore, it is imperative to explore maternal behaviors that may optimize the maternal-fetal environment leading to the delivery of full-term, healthy-weight neonates; thus, eliminating the racial/ethnic disparities in these adverse maternal and neonatal outcomes.

Over the past two decades, accumulating evidence demonstrates many maternal, fetal and neonatal benefits consequent to participation in recommended levels of exercise during pregnancy [5]. Women engaging in at least the recommended American College of Obstetricians and Gynecologists (ACOG) guidelines [6] (> 150 min per week or 500 MET∙min∙week^− 1^), experience healthy weight gain [7] and decreased risks of metabolic complications (e.g., gestational diabetes mellitus) [8, 9]. Moreover, neonates exposed to exercise in utero exhibit lower percent body fat [10], increased cardiovascular function [11, 12], and enhanced neuromotor skills [13]. With regard to delivery and birth outcomes, current evidence suggests that prenatal exercise does not increase the risks of preterm births or delivery of low-birthweight neonates and may lower risk of cesarean sections [14, 15]. Most of these studies, however, did not explore racial/ethnic differences in these outcomes. Limited studies examined these associations among NHB pregnant women and demonstrated a reduction in risk in these outcomes among NHB pregnant women [16, 17]. However, these studies did not employ rigorous study designs or measures of prenatal exercise. Thus, the effectiveness of prenatal exercise to close the gap in the racial/ethnic disparities in select delivery and birth outcomes between NHB and NHW pregnant women is unclear. As such, the purpose of this study was to determine the effects of a prenatal aerobic moderate-intensity exercise intervention on the association between maternal race/ethnicity and select delivery and birth outcomes between NHB and NHB white pregnant women. We hypothesized that for all outcomes, prenatal exercise would reduce the racial/ethnic disparities between NHB and NHW pregnant women and their neonates.

## Methods

### Study design

This study employed a secondary data analysis using data from a prospective randomized study (ENHANCED by Mom) focused on the influence of exercise type on infant outcomes throughout pregnancy [18]. Women were recruited from local obstetric clinics via brochures, flyers, word-of-mouth and social media in eastern North Carolina. All protocols were approved by the Institutional Review Board at East Carolina University and registered in clinicaltrials.gov (NCT03517293). Each eligible and interested participant read and signed a written informed consent prior to enrollment. Pregnant women were enrolled between 13 and 16 weeks of gestation and participated in the exercise intervention until delivery.

### Study population

Inclusion criteria for this study were healthy, low-risk pregnancy, defined as follows: 1) singleton pregnancy (< 16 weeks of gestation), 2) between 18 and 40 years of age, 3) pre-pregnancy body mass index (BMI) between 18.5 to 34.99 kg/m^2^, 4) physician clearance to participate in an exercise program, and 5) able to communicate fluently in English and be contacted via phone or email. Women were excluded from the study if they: 1) had pre-existing medical conditions (e.g., diabetes, hypertension) or conditions known to affect fetal development (e.g., systemic lupus erythematosus), 2) were taking medications known to affect fetal development or pregnancy outcomes, or 3) were using tobacco, alcohol, or other recreational drugs. If an interested participant met these criteria, then the informed consent was obtained prior to study enrollment.

### Randomization

Following study enrollment and prior to group randomization, participants completed a submaximal exercise treadmill test, conducted by trained research staff, to determine their individual aerobic capacity and calculate their target heart rate (THR) ranges for moderate-intensity aerobic exercise training. Peak oxygen consumption (VO_2peak_) was estimated via the modified Balke protocol previously validated and replicated for pregnant women by Mottola et al. (2006) [19]. After completing this test, participants were randomized via computerized sequencing (GraphPad software) to aerobic exercise or a non-exercising comparison group.

### Exercise intervention

Both the aerobic exercise and non-exercising stretching/breathing groups participated in one-on-one sessions, three times per week for at least 50 min. All participants began each session with a 5-min warm-up and ended with a 5-min cool-down. All participants tailored their program, choosing 3 days weekly to attend supervised sessions. All training sessions were supervised by a trained staff member. Resting heart rate (HR) and blood pressure were assessed before and after each session; HR (Polar FS2C HR monitor) and rating of perceived exertion (RPE) were monitored throughout exercise to maintain appropriate intensity. The aerobic training group exercised on upright, weight bearing aerobic machines (i.e. treadmill, elliptical, bicycle) of their choice meeting the ACOG guidelines of *150 min per week of moderate intensity* (40–59% VO_2peak_; 60–80% aerobic capacity, RPE of 12–14) exercise. The non-exercising control group attended low-intensity (< 40% VO_2peak_) sessions focused on the stretching of major muscle groups while incorporating appropriate breathing techniques. Supervised exercise and stretching/breathing sessions occurred at one of two university-affiliated gyms. HR monitors were worn to ensure that maternal HRs were in the appropriate range. Pregnancy MET∙min∙wk.^− 1^ [20] were quantified (frequency X duration of session) then multiplied by the MET (metabolic equivalent) level of their assigned group, established by the Compendium for Physical Activity [20]. The total for all weeks were summed and averaged for the pregnancy MET∙min∙wk.^− 1^ value.

### Exercise adherence

Exercise session attendance was tracked via an electronic record in REDCap and calculated by dividing the number of sessions attended by the total number of potential sessions within the participants’ gestational period. Participants were considered “exercise adherent” if their attendance was ≥80% of possible exercise sessions. Adherence to randomized groups throughout pregnancy was verified via completion of the modifiable physical activity questionnaire (MPAQ) at the end of pregnancy and after the birth of their child.

### Maternal measurements

Maternal demographic and pregnancy-related characteristics including age, gravida, pre-pregnancy weight and height, gestational diabetes mellitus status (yes or no) were abstracted from various sources including pre-screening eligibility questionnaires and electronic health records. Pre-pregnancy BMI was calculated using self-reported height and weight collected from the pre-screening eligibility questionnaire (16 weeks of gestation) via the following established equation: *BMI* = ((*weight* (*kg*)) ÷ ([*height* (*m*^2^)])). BMI classifications used were previously established [21]:18.5–24.9 kg/m^2^ was designated as normal, 25.0–29.9 kg/m^2^ was overweight, and 30.0–39.9 kg/m^2^ was obese class I and II.

### Neonatal birth measures

Delivery mode (cesarean section & standard vaginal delivery), gestational age in weeks, and birth weight in kilograms served as the primary outcomes of this study and were acquired from the electronic health records. Gestational age in weeks was determined using the date of their last menstrual period, unless unknown, then the first ultrasound scan during their soonest prenatal visit was used. Preterm birth was defined as a delivery at < 37 weeks of gestation. Low birthweight was defined as a neonate weighing < 2.5 kg at birth. The cut-offs for these outcomes were previously established by medical professionals and research and shown to be associated with poor health outcomes [22]. Neonatal circumferences (head & abdominal), length and sex were also collected from electronic health records.

### Statistical analysis

Between-group differences in maternal and neonatal descriptive characteristics were determined via Student’s t-tests and Pearson Chi-Square tests. Conditional distributions were evaluated, via residual plots, for the assumptions of linear and Poisson regression and were satisfied. The primary outcomes of this study were: birthweight (kg; continuous), gestational age (weeks; continuous), relative risks of low birthweight (LBW, < 2.5 or ≥ 2.5 kg), preterm (< 37 or ≥ 37 weeks), and delivery mode (vaginal or cesarean). Following, ANCOVA and Poisson regression models were performed to evaluate the effects of prenatal exercise on the association between race and pregnancy and birth outcomes. The main effects between race and the outcomes of interest were assessed first, followed by the effects of prenatal exercise (MET∙min∙week^− 1^). Next, the following covariates were considered where appropriate: gestational age, neonatal sex, pre-pregnancy BMI, maternal age, and gravida. Two sets of analyses were performed, 1) intention-to-treat (ITT), including all participants regardless of the exercise dose received and 2) per protocol, restricting the analytical sample to those attending ≥80% of total possible exercise sessions. Statistical analyses were performed using SAS, version 9.4 (Cary, NC). Statistical significance was determined a priori at *p* < 0.05.

## Results

### Participant recruitment and retention

Between 2015 and 2018, 188 pregnant women were assessed for eligibility. Of these, 173 were randomized to aerobic exercise (*n* = 107) or a non-exercising control (*n* = 66). Fifteen participants did not receive their assigned intervention due to group refusal (*n* = 14, all control) and miscarriage (n = 1) and 2 pregnant women were lost-to-follow-up consequent to leaving the geographical area, or no time for participation; 1 was excluded for drug use, and 3 had missing data on their electronic health record. Of the remaining 125 pregnant women (exercise = 58 and control = 67), there were 30 NHB (24.0%, 37) and 95 NHW. All women were healthy with no pregnancy complications. For the per protocol analysis, data from 15 mother: neonate pairs were excluded due to ‘non-adherence’ to the exercise intervention yielding the stated sample of 97 mother: neonate pairs.

For both the ITT and per protocol samples for EX vs CON comparisons, exercising women tended to be older (only significant for per protocol) with a lower pre-pregnancy BMI than non-exercising controls (Table 1); there were comparable racial/ethnic compositions between groups (ITT: NHW 17.2% vs 29.9%; per protocol: NHW 13.3% vs 29.9%). No other significant between-group differences were observed. Roughly 20% of women, in both groups, delivered via cesarean section and between 5 to 8% were diagnosed with GDM. A few significant differences were observed between NHW and NHB pregnant women (Table 2). Regardless of group allocation, average NHB pregnant women were obese and were less physically active than NHW women.

**Table 1:** Maternal Descriptive, Pregnancy and Delivery Outcomes by Intervention Group and Exercise Adherence

Characteristics	Intention-to-Treat			Per Protocol (≥ 80% adherence)		
	Aerobic (n = 58)	Control (n = 67)	p-value	Aerobic (***n*** = 30)	Control (n = 67)	p-value
**Demographics**						
Age (yr.)^a^	30.7 (4.0)	29.4 (4.3)	0.11	31.7 (3.2)	29.4 (4.3)	0.01
**Race/Ethnicity**						
NHB (n)^c^	17.2% [10]	29.9% [20]	0.10	13.3% [4]	29.9% [20]	0.13
**Education Level**^c, d^						
Post-Secondary	98.0% (49)	80.4% (45)	0.005	96.3% [26]	80.4% (45)	0.09
Pre-Pregnancy BMI^a^	24.7 (4.8)	27.0 (5.8)	0.02	23.4 (3.5)	27.0 (5.8)	0.0003
**Pregnancy**						
Gravida^b^	1.5 (1.0, 5.0)	1.0 (1.0, 6.0)	0.81	1.0 (1.0, 4.0)	1.0 (1.0, 6.0)	0.69
GDM (n)^c^	5.3% [3]	7.6% [5]	0.72	6.7% [2]	7.6% [5]	1.00
Gestational Weight Gain (lb)	30.6 (12.5)	28.9 (13.1)	0.66	31.1 (12.4)	28.9 (13.1)	0.48
**Delivery**						
Cesarean (%)^c^	21.1% [12]	23.8% [16]	0.64	20.0% [6]	23.8% [16]	0.79

Note: Means (SD), medians (range) and proportions are reported and ^a^Student’s t-test, ^b^Wilcoxon Rank Sum tests and ^c^Fisher’s Exact tests were performed, respectively. *NHB* Non-Hispanic black; *BMI* body mass index; *GDM* gestational diabetes mellitus. Gestational weight gain was calculated by difference in self-reported pre-pregnancy weight and delivery weight, collected via medical records. ^d^Nineteen participants had missing data for level of education

**Table 2:** Maternal Descriptive, Pregnancy and Delivery Outcomes by Maternal Race

Characteristics	NHW (***n*** = 95)	NHB (n = 30)	p-value
**Demographics**			
Age (yr.)^a^	30.2 (3.5)	29.3 (5.8)	0.29
Pre-Pregnancy BMI^a^	25.2 (5.2)	28.5 (5.7)	0.004
**Education Level**^c, d^			
Post-Secondary (%, n)	96.5% (82)	57.1% [12]	< 0.0001
**Activity Level**^a^ METs (ml O_2_·kg^−1^·min^− 1^)	393.0 (0.0, 1225.0)	163.9 (0.0, 942.0)	0.002
**Pregnancy**			
Gravida^b^	1.0 (1.0, 5.0)	2.0 (1.0, 6.0)	0.34
GDM (n)^c^	7.5% [7]	3.3% [1]	0.42
Gestational Weight Gain (lb)	30.1 (12.1)	28.7 (15.3)	0.66
**Delivery**			
Cesarean Section (n)^c^	21.7 (21)	26.7 (8)	0.62

Note: Means (SD), medians (range) and proportions are reported and ^a^Student’s t-test, ^b^Wilcoxon Rank Sum tests and ^c^Fisher’s Exact tests were performed, respectively. BMI = body mass index (kg/m^2^); *GDM* gestational diabetes mellitus. *METs* metabolic equivalents expressed in milliliters of oxygen per kilogram per minute. *NHW* Non-Hispanic White; *NHB* Non-Hispanic Black. ^d^Nineteen participants had missing data for level of education

There were no significant between-group differences, for EX vs CON comparisons, observed for any neonatal outcomes in both the ITT and per protocol samples (Table 3). Conversely, significant differences in birth outcomes were found between neonates born to NHW and NHB pregnant women (Table 4). On average, neonates born to NHB pregnant women were lighter (3.0 kg vs 3.4 kg; *p* = 0.001) with smaller head (33.0 cm vs 34.3 cm; *p* = 0.01) and abdominal (29.7 cm vs 31.8 cm; *p* = 0.04) circumferences, and were shorter in length (0.48 m vs 0.50 m; *p* = 0.004). No other significant differences were found between neonates of NHW and NHB pregnant women.

**Table 3:** Neonatal Characteristics and Birth Outcomes (mean or median, SD or range) by Intervention Group and Exercise Adherence

Characteristics	Intention-to-Treat			Per Protocol (≥ 80% adherence)		
	Aerobic (n = 58)	Control (n = 67)	***p***-value	Aerobic (n = 30)	Control (n = 67)	p-value
**Sex**						
Male (n)^c^	50.0% [29]	45.6% [31]	0.68	50.0% [15]	45.6% [31]	0.83
**Length of Gestation (weeks)**^b^	39.6 (30.5, 41.9)	39.3 (23.7, 41.3)	0.53	39.7 (34.0, 41.9)	39.3 (23.7, 41.3)	0.39
Preterm (n)^c^	13.8% [8]	12.1	1.00	13.3	12.1	1.00
**Size at Birth**						
Birthweight (kg)^a^	3.4 (0.7)	3.3 (0.6)	0.63	3.3 (0.7)	3.3 (0.6)	0.96
Low Birthweight (n)^c^	13.8% [8]	9.0% [6]	0.49	13.8% [8]	9.0% [6]	0.49
Birth Length (m)^b^	0.50 (0.35, 0.56)	0.50 (44.5, 0.54)	0.46	0.49 (0.35, 0.53)	0.50 (44.5, 0.54)	0.45
Head Circumference (cm)^b^	34.5 (26.0, 38.1)	33.7 (28.0, 45.0)	0.32	34.5 (28.5, 38.1)	33.7 (28.0, 45.0)	0.46
Abdominal Circumference (cm)^b^	31.5 (23.5, 35.6)	31.0 (22.0, 37.5)	0.49	31.1 (23.5, 35.6)	31.0 (22.0, 37.5)	0.78

Note: Means (SD), medians (range), and proportions are reported and ^a^student’s t-test, ^b^Wilcoxon Rank Sum, ^c^Fisher’s exact tests were performed, respectively

**Table 4:** Neonatal Birth Outcomes and Characteristics (mean or median, SD or range) by Maternal Race

Characteristics	NHW (n = 95)	NHB (n = 30)	p-value
**Sex**			
Male (n)^c^	48.9% (46)	40.0% [12]	0.41
**Length of Gestation (weeks)**	39.5 (34.0, 41.9)	39.1 (23.7, 41.3)	0.14
Preterm (n)^c^	8.5% [8]	20.0% [6]	0.10
**Size at Birth**			
Birthweight (kg)^a^	3.4 (0.6)	3.0 (0.7)	0.001
Low Birthweight (n)^c^	8.4% [8]	16.7% [5]	0.30
Birth Length (m)^b^	0.50 (0.35, 0.56)	0.48 (0.39, 0.56)	0.004
Head Circumference (cm)^b^	34.3 (28.5, 45.0)	33.0 (26.0, 36.0)	0.01
Abdominal Circumference (cm)^b^	31.8 (23.5, 36.0)	29.7 (22.0, 37.5)	0.04

Note: Means (SD), medians (range) and proportions are reported and ^a^Student’s t-test, ^b^Wilcoxon Rank Sum tests and ^c^Fisher’s Exact tests were performed, respectively. *NHW* Non-Hispanic White; *NHB* Non-Hispanic Black

Results for the effects of maternal race on delivery and birth outcomes and the independent effect of prenatal exercise for the ITT analyses are presented in Table 5. Adjusted regressions showed that, after controlling for maternal pre-pregnancy BMI, maternal race was significantly associated with neonatal birthweight, where NHB pregnant women delivered neonates weighing nearly 0.5 kg less (β = − 0.43, 95%CI: − 0.68, − 0.18, *p* = 0.001) than neonates born to NHW pregnant women. Prenatal exercise attenuated this association with reductions observed for the regression coefficient (β: − 0.43 vs − 0.39 kg), representing a 0.04 kg increase in neonatal birthweight. No other statistically significant associations were found in the ITT sample for the association between maternal race and delivery and birth outcomes or the effects of prenatal exercise on these relationships.

**Table 5:** Intention-to-Treat: Linear, Median and Poisson Regression Coefficients (β, 95%CI, *p*-value) for the Effects of Prenatal Exercise on Race and Delivery and Birth Outcomes

Characteristics	Model (Race + covariate [s])			Adjusted (Model 1 + Exercise)		
	β or RR^**b**^	95%CI	p-value	β or RR^**b**^	95%CI	p-value
**Delivery**						
Gestational Age^a^	− 0.67	− 1.40, 0.06	0.07	− 0.48	− 1.2, 0.22	0.18
Preterm (%)^b^	2.33	0.99, 8.94	0.053	2.23	0.70, 7.04	0.17
Cesarean Section (%)^b^	1.04	0.72, 1.50	0.83	1.04	0.71, 1.53	0.84
**Birth Outcomes**						
Birthweight (kg) ^c^	−0.43	−0.68, − 0.18	0.001	− 0.39	− 0.65, − 0.13	0.004
Low Birthweight (%)^b^	2.24	0.71, 7.05	0.17	2.02	0.59, 6.86	0.26

Note: Model-specific covariates included 1) *gestational age:* pre-pregnancy BMI, MET∙min∙week^−1^; 2) *preterm:* pre-pregnancy BMI, MET∙min∙week^− 1^; 3) *cesarean section:* MET∙min∙week^− 1^; 4) *birthweight:* MET∙min∙week^− 1^; and 5) *low birthweight:* MET∙min∙week^− 1^. ^a^ Median regression was performed and the respective βs were reported. ^b^ Poisson regression was performed, and the respective relative risks are reported. ^c^ Linear regression was performed and the respective βs were reported

For the per protocol sample, the results for the effects of maternal race on delivery and birth outcomes and independent effects of exercise are shown in Table 6. Similar to the ITT results, maternal race was significantly associated with neonatal birthweight with NHB pregnant women delivering neonates nearly 0.5 kg lighter compared to neonates born to NHW pregnant women (β = − 0.41, 95%CI: − 0.69, − 0.13, *p* = 0.005). Prenatal exercise attenuated this relationship, reducing the differences in neonatal birthweight between NHB and NHW pregnant women by 0.04 kg (β: − 0.43 vs − 0.39). No other statistically significant effects were found for maternal race and delivery and birth outcomes or the effect of prenatal exercise on these associations.

**Table 6:** Per Protocol: Linear, Median and Poisson Regression Coefficients (β, 95%CI, p-value) for the Effects of Prenatal Exercise on Race and Delivery and Birth Outcomes

Characteristics	Model (Race + covariate [s])			Adjusted (Model 1 + Exercise)		
	β or RR^b^	95%CI	p-value	β	95%CI	p-value
**Delivery**						
Gestational Age^a^	−0.73	−1.55, 0.09	0.08	−0.69	−1.5, 0.14	0.11
Preterm (%) ^b^	2.14	0.68, 6.75	0.19	1.98	0.58, 6.76	0.27
Cesarean Section (%) ^b^	1.11	0.74, 1.67	0.60	1.12	0.73, 1.73	0.61
**Birth Outcomes**						
Birthweight (kg)^c^	−0.41	−0.69, −0.13	0.005	−0.39	− 0.69, − 0.09	0.01
Low Birthweight (%) ^b^	2.40	0.64, 8.94	0.19	2.20	0.53, 9.13	0.29

Note: Model-specific covariates included 1) *gestational age:* pre-pregnancy BMI, MET∙min∙week^−1^; 2) *preterm:* pre-pregnancy BMI, MET∙min∙week^− 1^; 3) *cesarean section:* MET∙min∙week^− 1^; 4) *birthweight:* MET∙min∙week^− 1^; and 5) *low birthweight:* MET∙min∙week^− 1^. ^a^ Median regression was performed and the respective βs were reported. ^b^ Poisson regression was performed, and the respective relative risks are reported. ^c^ Linear regression was performed and the respective βs were reported

## Discussion

The purpose of this study was to examine the effects of prenatal exercise on the association between maternal race/ethnicity and select delivery and birth outcomes. The major findings of this study were 1) maternal exercise performed through the 2nd and 3rd trimesters of pregnancy partially attenuated the relationship between maternal race/ethnicity and neonatal birthweight, such that the racial/ethnic differences in neonatal birthweight between NHB and NHW pregnant women was reduced, 2) prenatal exercise did not appear to significantly (statistically) affect the relationship between maternal race/ethnicity and gestational age or risks of preterm births, cesarean sections and the delivery of low-birthweight neonates.

A novel aspect of this study was that participation in exercise in the latter two-thirds of pregnancy positively attenuated the relationship between maternal race/ethnicity and neonatal birthweight. Specifically, this study observed that the documented disparities in neonatal birthweight between NHB and NHW pregnant women, where NHB pregnant women delivered lighter neonates (3.4 kg vs 3.0 kg), was reduced by 0.04 kg (40 g). While apparently “small” in magnitude, a 40 g increase neonatal birthweight, accounts for 20% of the consistently documented 200 g difference in birthweight of neonates born to NHB and NHW pregnant women [23, 24]. In a previous study, a 14-g increase in neonatal birthweight, consequent to increased iron intake, was attributed to the observed risk reduction in low-birthweight neonates [25]. While that study’s association different from the present study, it highlights the possible clinical significance of smaller increments in birthweight on neonatal health and wellbeing.

The observed racial disparities in neonatal birthweight between NHB and NHW pregnant may be attributed to different factors, some of which may be influenced by prenatal exercise. Studies report higher levels of perceived stress [26] and chronic disease risk factors such as hypertension [27], endothelial dysfunction [28], inflammation [29] and obesity etc. [30] among NHB women during pregnancy. These risk factors negatively affect fetal growth and are posited to result in lower-birthweight infants. Importantly, while limited studies exist in the pregnant population, several studies in non-pregnant populations consistently show that chronic exercise reduces perceived stress [31], serum cortisol levels [31], endothelial function [32, 33], inflammation [34] and obesity-related health consequences [35]. We speculate that these positive effects of exercise may mitigate the negative effects of the aforementioned risk factors during pregnancy on fetal growth, subsequently reducing the disparities in neonatal birthweight between NHB and NHW, as observed in the present study. While both NHB and NHW pregnant women in this study were exposed to the same exercise training regimen, the NHB pregnant were on average classified as obese and less physically active than their NHW counterparts. Thus, it is possible that the NHB pregnant women exhibited a stronger effect of prenatal exercise given the aforementioned co-morbidities associated with obesity and low levels of physical activity.

Moreover, another explanation for the decrease in the race/ethnic disparity in neonatal birthweight is the increased cardiac output and decreased peripheral resistance consequent to chronic exercise training. Clapp et al. (1992, 2000, 2002, 2003), Pivarnik et al. (1994) and Jackson et al. (1995) consistently demonstrated positive effects of chronic prenatal exercise on the maternal cardiovascular system, feto-placental growth and increased neonatal birthweight [36–41]. Importantly, these cardiovascular adaptations to exercise may augment the naturally occurring changes to the maternal cardiovascular system and placental blood flow and growth [39]. With this evidence, we posit that these adaptations enhanced fetal nutrient delivery and increased neonatal birthweight, possibly more so among NHB women during pregnancy. In the present study, we acknowledge the limitation that these hypothesized adaptations of prenatal exercise were not measured. However, no scientific studies, to our knowledge exist in the pregnant population that examine the effects of prenatal exercise on the racial/ethnic disparities in fetal and neonatal growth and the associated physiological factors. As such, these speculations are drawn from the available evidence conducted in pregnant and non-pregnant populations.

Unexpectedly, this study did not observe a significant effect of prenatal exercise on the relationship between maternal race/ethnicity and gestational age or the risks of preterm births, cesarean sections or low-birthweight neonates. While non-statistically significant, the attenuating effects of prenatal exercise on the relationship between maternal race/ethnicity and gestational age (β = − 0.67, *p* = 0.07 to β = − 0.48, *p* = 0.17) and risk of low-birth infants (RR = 2.42, p = 0.17 to RR = 2.02, *p* = 0.26), were in the hypothesized direction. A potential explanation for these null findings may be the disproportion of NH black pregnant women in the non-exercising control group. Moreover, in this study, NHB pregnant women were less active compared to NHW pregnant women (393.0 vs 163.9 MET∙min∙week^− 1^) potentially reducing the effect of prenatal exercise on the relationships between maternal race/ethnicity and these adverse outcomes. Another explanation for the null observations is the small proportion of NHB women and lower prevalence of cesarean sections, preterm births and low-birthweight infants precluding our ability to detect an attenuating effect of prenatal exercise. In addition, a larger sample would allow for the evaluation of a potential moderating effect of prenatal exercise on the maternal racial/ethnic disparities in these outcomes, providing information on possible differential effects among various minority groups.

### Strengths and limitations

There are several strengths of the present study. First, to the best of our knowledge, this is the first study to evaluate the effects of recommended levels of maternal exercise during the 2nd and 3rd trimesters of pregnancy on racial/ethnic disparities in delivery and birth outcomes. Our findings show a potentially, positive effect of prenatal exercise, which appeared to reduce the disparities in neonatal birthweight between NH black and NH white women. This finding extends the growing evidence base for the documented benefits of prenatal exercise, emphasizing the important of exercise throughout pregnancy. Second, this study used data from a previously conducted a prospective, supervised exercise intervention study increasing the robustness of our exercise exposure. In addition to the strengths of this study, we acknowledge some limitations. First, our sample included a small proportion of NH black women, which increased the difficulty of detecting the effects of prenatal exercise on the previously documented disparities in delivery and birth outcomes assessed in this study. Second, the unbalanced sample sizes between NHW and NHB pregnant women potentially reduced our statistical power to given the likely increased variability in the small NHB group. Third, the overall sample size was insufficient for more detailed analyses including mediation and moderation. Post-hoc power analyses showed the statistical power and cohen’s d effect size (훃, ES) for the following outcomes: preterm birth (63%, 0.27), low birthweight (57%, 0.24), cesarean section (33%, 0.04), and birthweight (92%, 0.2). Currently, no statistical power analysis method exists for quantile regression, thus the statistical power for gestational age cannot be calculated. However, given the non-significant findings, this analysis and others, the analyses were underpowered, with the exception of birthweight.

## Conclusion

Based on these findings, prenatal exercise may decrease the likelihood of NH Black women delivering with more adverse outcomes. Specifically, prenatal exercise may reduce the racial/ethnic disparities in neonatal birthweight, possibly increasing the birthweight among NH Black women. Importantly, birthweight is a predictor of future health trajectories of neonates. Prenatal exercise did not exert measurable attenuating effects on gestational age or risks of preterm birth and low-birthweight neonates, although associations were in the hypothesized direction. Further research is needed to elucidate the effects of prenatal exercise on the racial/ethnic disparities in birth outcomes, specifically studies requiring larger, racial/ethnic diverse samples, measurements of metabolic biomarkers, and more comprehensive analyses to examine potential underlying mechanisms.

## Data Availability

De-identified data is available upon request to the lead PI, Dr. Linda May, mayl@ecu.edu.

## References

[1] Petersen EE, Davis NL, Goodman D, Cox S, Syverson C, Seed K, et al. Racial/ethnic disparities in pregnancy-related deaths - United States, 2007-2016. Morb Mortal Wkly Rep. 2019;68(35):762–5. 10.15585/mmwr.mm6835a3 PMC6730892 31487273

[2] Martin JA, Hamilton BE, Osterman MJ, Driscoll AK (2019). Birth: final data for 2018. 2019. Nat Vital Stats Report.

[3] Barker DJP, Osmond C, Law CM (1989). The intrauterine and early postnatal origins of cardiovascular disease and chronic bronchitis. J Epidemiol Community Health.

[4] Barker DJP (2000). In utero programming of cardiovascular disease. Theriogenology..

[5] Impact of physical activity during pregnancy and postpartum on chronic disease risk. Med Sci Sports Exerc. 2006;38(5):989–1006. 10.1249/01.mss.0000218147.51025.8a. 10.1249/01.mss.0000218147.51025.8a 16672855

[6] American College of Obstetricians and Gynecologists. Physical activity and exercise during pregnancy and the postpartum period. Obstet Gynecol. 2020;135:e178–88. 10.1097/AOG.0000000000003772 32217980

[7] McDonald SM, Liu J, Wilcox S, Lau EY, Archer E (2016). Does dose matter in reducing gestational weight gain in exercise interventions? A systematic review of literature. J Sci Med Sport.

[8] Davenport MH, Ruchat S-M, Poitras VJ, Jaramillo Garcia A, Gray CE, Barrowman N, et al. Prenatal exercise for the prevention of gestational diabetes mellitus and hypertensive disorders of pregnancy: a systematic review and meta-analysis. Br J Sports Med. 2018 Nov 1;52(21):1367–75. 10.1136/bjsports-2018-099355. 10.1136/bjsports-2018-099355 30337463

[9] Clark E, Isler C, Strickland D, McMillan AG, Fang X, Kuehn D (2019). Influence of aerobic exercise on maternal lipid levels and offspring morphometrics. Int J Obes.

[10] McDonald SM, Isler C, Newton E, Kuehn D, Kelley G, Chasan-Taber L, et al. The effects of moderate intensity prenatal aerobic exercise on neonatal morphometry: a randomized controlled trial. Birth Defects. *In press*.

[11] May LE, Scholtz SA, Suminski R, Gustafson KM (2014). Aerobic exercise during pregnancy influences infant heart rate variability at one month of age. Early Hum Dev.

[12] May LE, Suminski RR, Langaker MD, Yeh HW, Gustafson KM (2012). Regular maternal exercise dose and fetal heart outcome. Med Sci Sports Exerc.

[13] McMillan AG, May LE, Gaines GG, Isler C, Kuehn D (2019). Effects of aerobic exercise during pregnancy on 1-nonth infant neuromotor skills. Med Sci Sports Exerc.

[14] Tinloy J, Chuang CH, Zhu J, Pauli J, Kraschnewski JL, Kjerulff KH (2014). Exercise during pregnancy and risk of late preterm birth, cesarean delivery, and hospitalizations. Womens Health Issues.

[15] Di Mascio D, Magro-Malosso ER, Saccone G, Marhefka GD, Berghella V (2016). Exercise during pregnancy in normal-weight women and risk of preterm birth: a systematic review and meta-analysis of randomized controlled trials. Am J Obstet Gynecol.

[16] Orr ST, James SA, Garry J, Newton E (2006). Exercise participation before and during pregnancy among low-income, urban, black women: the Baltimore preterm birth study. Ethn Dis.

[17] Finch BK, Frank R, Hummer RA (2000). Racial/ethnic disparities in infant mortality: the role of behavioral factors. Soc Biol.

[18] Moyer C, Livingston J, Fang X, May LE (2015). Influence of exercise mode on pregnancy outcomes: ENHANCED by mom project. BMC Pregnancy Childbirth.

[19] Mottola MF, Davenport MH, Brun CR, Inglis SD, Charlesworth S, Sopper MM (2006). VO2peak prediction and exercise prescription for pregnant women. Med Sci Sports Exerc.

[20] Ainsworth BE, Haskell WL, Herrmann SD, Meckes N, Bassett DRJ, Tudor-Locke C (2011). Compendium of physical activities: a second update of codes and MET values. Med Sci sports Exerc.

[21] Garrow J, Webster J (1985). Quetelet’s index (W/H2) as a measure of fatness. Int J Obes.

[22] Quinn J-A, Munoz FM, Gonik B, Frau L, Cutland C, Mallett-Moore T, et al. Preterm birth: case definition & guidelines for data collection, analysis, and presentation of immunisation safety data. Vaccine. 2016;34(49):6047–56. 10.1016/j.vaccine.2016.03.045. 10.1016/j.vaccine.2016.03.045 PMC5139808 27743648

[23] David RJ, Collins JW (1997). Differing birth weight among infants of U.S.-born blacks, African-born blacks, and U.S.-born whites. N Engl J Med.

[24] Alexander K (1999). Himes, Mor, Goldenberg. Racial differences in birthweight for gestational age and infant mortality in extremely-low-risk US populations. Paediatr Perinat Epidemiol.

[25] Haider BA, Olofin I, Wang M, Spiegelman D, Ezzati M, Fawzi WW. Anaemia, prenatal iron use, and risk of adverse pregnancy outcomes: systematic review and meta-analysis. BMJ. 2013;346(3). 10.1136/bmj.f3443. 10.1136/bmj.f3443 PMC3689887 23794316

[26] Dominguez TP, Schetter CD, Mancuso R, Rini CM, Hobel C (2005). Stress in African American pregnancies: testing the roles of various stress concepts in prediction of birth outcomes. Ann Behav Med.

[27] Ghosh G, Grewal J, Mannisto T, Mendola P, Chen Z, Xie Y (2014). Racial/ethnic differences in pregnancy-related hypertensive disease in nulliparous women. Eth Dis.

[28] Mata-Greenwood E, Chen D-B (2008). Racial differences in nitric oxide-dependent vasorelaxation. Reprod Sci.

[29] Picklesimer AH, Jared HL, Moss K, Offenbacher S, Beck JD, Boggess KA (2008). Racial differences in C-reactive protein levels during normal pregnancy. Am J Obstet Gynecol.

[30] Snowden JM, Mission JF, Marshall NE, Quigley B, Main E, Gilbert WM, et al. The impact of maternal obesity and race/ethnicity on perinatal outcomes: independent and joint effects. Obesity. 2016;24(7):1590–8. 10.1002/oby.21532. 10.1002/oby.21532 PMC4925263 27222008

[31] Rimmele U, Zellweger BC, Marti B, Seiler R, Mohiyeddini C, Ehlert U, et al. Trained men show lower cortisol, heart rate and psychological responses to psychosocial stress compared with untrained men. Psychoneuroendocrinology. 2007;32(6):627–35. 10.1016/j.psyneuen.2007.04.005. 10.1016/j.psyneuen.2007.04.005 17560731

[32] Higashi Y, Yoshizumi M (2004). Exercise and endothelial function: role of endothelium-derived nitric oxide and oxidative stress in healthy subjects and hypertensive patients. Pharmacol Ther.

[33] Kemi OJ, Haram PM, Wisløff U, Ellingsen Ø (2004). Aerobic fitness is associated with cardiomyocyte contractile capacity and endothelial function in exercise training and detraining. Circulation..

[34] Beavers KM, Brinkley TE, Nicklas BJ (2010). Effect of exercise training on chronic inflammation. Clin Chim Acta.

[35] Church TS, Blair SN, Cocreham S, Johannsen N, Johnson W, Kramer K (2011). Effects of aerobic and resistance training on hemoglobin A1c levels in patients with type 2 diabetes. JAMA.

[36] Clapp JF III. Long-term outcome after exercising throughout pregnancy: fitness and cardiovascular risk. Am J Obstet Gynecol. 2008;199(5):489.e1–6. 10.1016/j.ajog.2008.05.006 PMC2650435 18667190

[37] Clapp JF, Kim H, Burciu B, Lopez B (2000). Beginning regular exercise in early pregnancy: effect on fetoplacental growth. Am J Obstet Gynecol.

[38] Clapp JF, Kim H, Burciu B, Schmidt S, Petry K, Lopez B (2002). Continuing regular exercise during pregnancy: effect of exercise volume on fetoplacental growth. Am J Obstet Gynecol.

[39] Clapp Iii JF. The effects of maternal exercise on fetal oxygenation and feto-placental growth. Eur J Obst Gynecol Reprod Biol. 2003;110(Supplement):S80–5. 10.1016/s0301-2115(03)00176-3 12965094

[40] Pivarnik JM, Mauer MB, Ayres NA, Kirshon B, Dildy GA, Cotton DB (1994). Effects of chronic exercise on blood volume expansion and hematologic indices during pregnancy. Obstet Gynecol.

[41] Jackson MR, Gott P, Lye SJ, Ritchie JW, Clapp JF (1995). The effects of maternal aerobic exercise on human placental development: placental volumetric composition and surface areas. Placenta..

